# Engineered HSV vector achieves safe long-term transgene expression in the central nervous system

**DOI:** 10.1038/s41598-017-01635-1

**Published:** 2017-05-04

**Authors:** Gianluca Verlengia, Yoshitaka Miyagawa, Selene Ingusci, Justus B. Cohen, Michele Simonato, Joseph C. Glorioso

**Affiliations:** 1 Section of Pharmacology, Department of Medical Sciences, and National Institute of Neuroscience, University of Ferrara, 44121 Ferrara, Italy; 2 Division of Neuroscience, University Vita-Salute San Raffaele, 20132 Milan, Italy; 3 Department of Microbiology and Molecular Genetics, University of Pittsburgh School of Medicine, Pittsburgh, PA 15219 USA; 4 Department of Biochemistry and Molecular Biology, Nippon Medical School, Tokyo, Japan

## Abstract

Previously we reported a new series of highly defective herpes simplex virus type 1 (HSV-1) vectors that were functionally devoid of all viral immediately early (IE) genes, resulting in virtual absence of viral gene expression. Nevertheless, a reporter gene cassette inserted into the vector flanked by boundary elements from the viral latency locus showed high, persistent reporter gene activity in non-neuronal cells while an independent expression cassette inserted into a deleted ICP4 locus remained almost silent. In contrast to non-neuronal cells, we show here that the ICP4 locus cassette permitted robust reporter gene expression in a diversity of neurons following stereotactic injection of different rat brain regions; transgene expression in the hippocampus lasted up to 6 months and was essentially restricted to neurons. No evidence of neuronal cell toxicity or induction of inflammatory cell infiltrates was observed. An independent reporter gene cassette located in an intergenic region remained silent, indicating that the transgene promoter and/or insertion site are critical for sustained expression. These findings suggest the suitability of this vector for therapeutic intervention into diseases of the central nervous system that require the expression of large and/or multiple therapeutic transgenes.

## Introduction

Neurological diseases have an enormous medical and social impact and are without effective treatments. Gene therapies provide a potential means of genetic intervention but, in most cases, treatment requires the availability of high capacity non-integrating gene transfer tools that provide durable, regulated expression of large or multi-gene cassettes in specific neuronal cell subpopulations^1^. In addition, many diseases are autosomal dominant requiring either knock out or knock down of the mutant gene product and either repair or complementing gene addition. We suggest that HSV vectors provide the best opportunity to meet these complex demands since HSV vectors can accommodate large inserts and are well adapted for life-long persistence in neurons as non-integrating, circular episomes.

We recently created a safe, high capacity recombinant HSV vector that is highly stable, unable to replicate, but capable of persisting in human primary cells without evidence of cytotoxicity at very high multiplicities of infection^2^. This vector expresses extremely low levels of viral lytic genes that can be detected only by RT-PCR, a result of functional deletion of all immediate early (IE) genes. The reiterated joint region separating the unique long (U_L_) and unique short (U_S_) genome components is also removed, providing a total of ~25 kb of space for transgene insertion. The challenge has been to further engineer this vector to provide durable transgene expression in both neuronal and non-neuronal cell types since transgenes are rapidly silenced in the absence of expression of one of the IE genes, ICP0, due to heterochromatin formation^3, 4^. We have recently overcome genome silencing through the application of moveable genetic elements from the latency-associated transcript (LAT) locus. These LAT locus-associated elements allow expression of non-viral transgene cassettes from the LAT or intergenic loci in non-neuronal cells without global activation of the viral genome^2^.

Here, we report on a vector design that allows robust long-term transgene expression in neurons of the brain from a non-latency-related locus. Transgene cassette replacement of the essential ICP4 IE gene provided transgene expression for at least 6 months in rat hippocampus and transgene activity was readily documented in a variety of other brain regions that included caudate putamen, substantia nigra, and cortex. While this genomic region is normally transcriptionally silent in neurons, changes in vector genome organization and the use of a foreign promoter appeared to contribute to high transcriptional activity at this site. Vector inoculation at high doses in multiple brain regions did not induce neuronal damage or attract an inflammatory infiltrate. This is the first HSV vector capable of long-term innocuous transgene expression in the brain and offers a new, highly engineered prototype vector that can now be applied to brain gene therapies where large or multiple transgene cassettes are required to achieve a therapeutic outcome.

## Results

### Vector design

**Figure 1:**
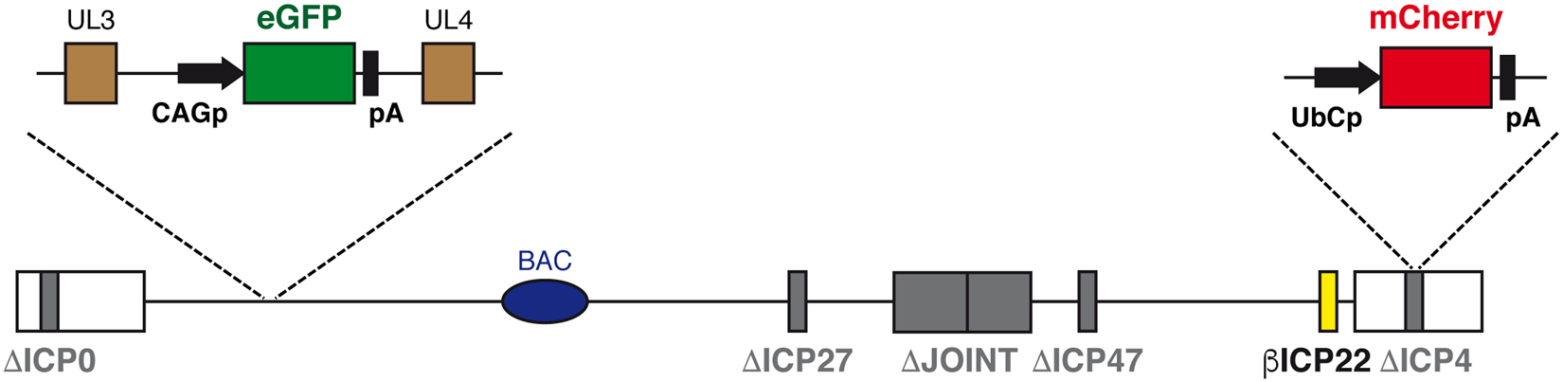
Graphic map of the JΔNI6 vector genome. The vector is deleted for the joint region, including the ICP47 promoter and translation initiation codon, and for the ICP0, ICP4 and ICP27 IE genes (Δ; gray boxes). Additionally, the ICP22 IE gene is converted to early-expression kinetics (β; yellow) by promoter TAATGARAT deletion. The vector hosts two expression cassettes for reporter genes: eGFP (green) between the U_L_3 and U_L_4 genes and mCherry (red) in the deleted terminal ICP4 locus. The eGFP gene is driven by the CAG promoter (CAGp), while the mCherry gene is driven by the ubiquitin promoter (UbCp). The BAC elements, including a chloramphenicol-resistance gene and β-galactosidase expression cassette, are located between *loxP* sites in the U_L_37-U_L_38 intergenic region.

The genome structure of the previously described JΔNI6GFP vector employed in this study, referred to here as JΔNI6, is shown in Fig. 1 ^2^. In this vector, the joint region, the ICP0, ICP4 and ICP27 IE genes, and the promoter and start codon of the ICP47 IE gene are deleted. In addition, the ICP22 IE gene is converted to early (β) expression kinetics by deletion of VP16-responsive promoter elements. The vector contains two expression cassettes for reporter genes: the enhanced green fluorescence protein (eGFP) gene inserted between the U_L_3 and U_L_4 genes under control of the CAG promoter, a strong synthetic promoter frequently used to drive high levels of transgene expression in mammalian cells; and the mCherry gene driven by the ubiquitin C (UbC) promoter inserted into the deleted ICP4 locus in the right terminal repeat. Bacterial artificial chromosome (BAC) genes allowing viral genome replication in bacteria, a chloramphenicol-resistance gene, and a β-galactosidase expression cassette are located between *loxP* sites in the U_L_37-U_L_38 intergenic region (Fig. 1). The genome also contains 2 mutations in the glycoprotein B (gB) gene previously shown to accelerate infection^5^.

### Transgene expression

**Figure 2:**
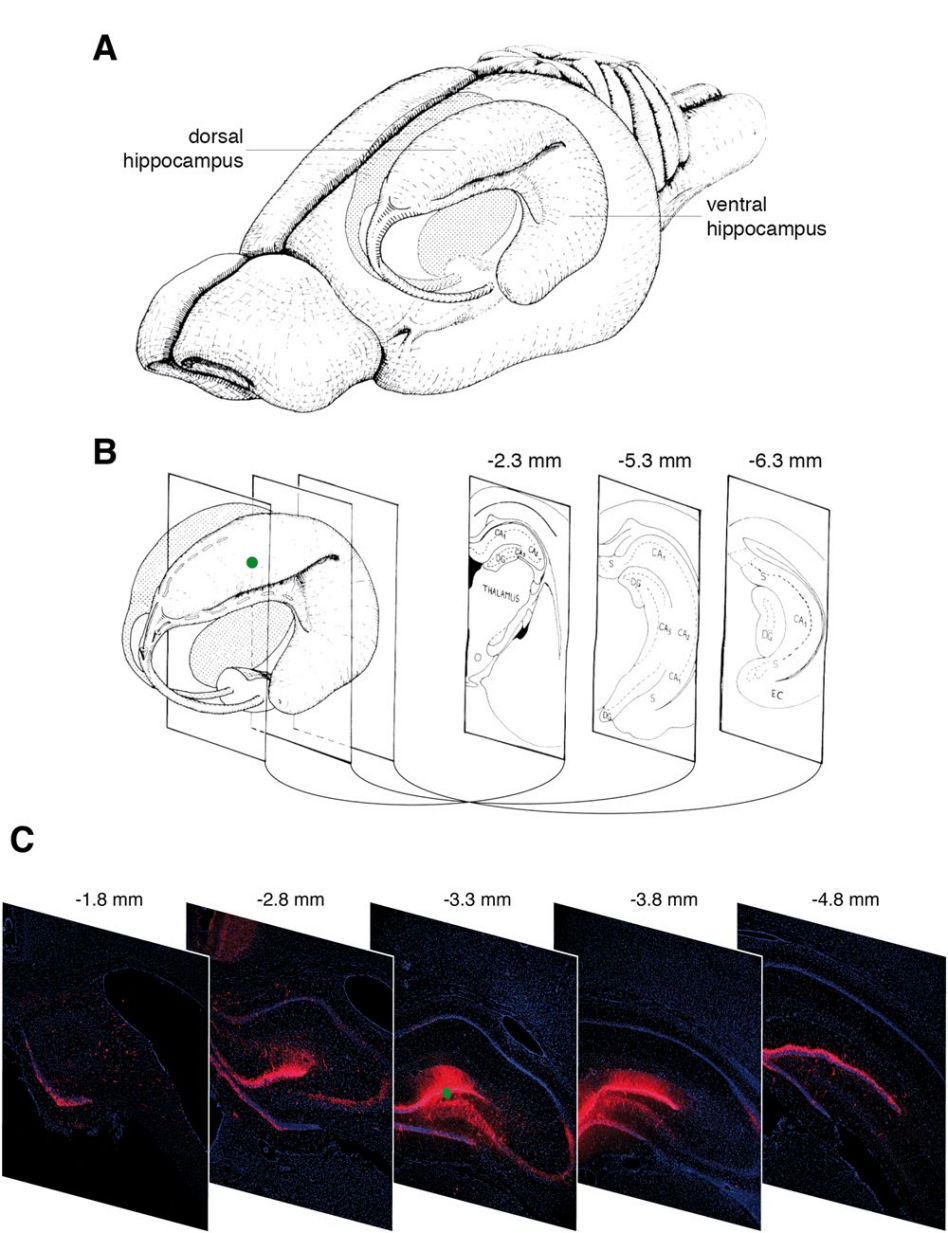
Distribution of cells expressing the mCherry reporter gene. (**A**) Drawing of the rat brain showing the three-dimensional organization of the hippocampus. (**B**) Coronal sections across the hippocampus. Three coronal sections through the hippocampus are shown at the right, including their anteroposterior coordinates relative to bregma. At the left, the green dot indicates the site of JΔNI6 injection. (**C**) Representative images of coronal sections from animals killed 2 months after JΔNI6 injection. Five coronal sections through the hippocampus are shown with their anteroposterior coordinates relative to bregma. Native mCherry fluorescence in red; nuclei stained with DAPI in blue. The green dot in the central image indicates the site of JΔNI6 injection. Panels A and B are adapted from Fig. 1 of Amaral and Witter^23^ with permission from Elsevier.

We first monitored transgene expression *in vivo* following injection of the dentate gyrus of the dorsal hippocampus with 2 μl of a solution containing 1 × 10^5^ plaque-forming units (pfu) of JΔNI6, corresponding to ~2.5 × 10^8^ genome copies (gc) (Fig. 2A,B). Diffusion of the vector was estimated by analyzing the presence of mCherry-positive cells in a broad span of coronal sections prepared at multiple levels, anterior to posterior of the injection site, from animals sacrificed 2 months after vector injection. We detected mCherry fluorescence in a range of more than 3 mm (Fig. 2C). It is likely that this is an underestimation of vector diffusion, because (i) transgene expression may occur in only a subset of infected cells and (ii), more importantly, we examined in this experiment intrinsic mCherry fluorescence, which can be detected only in cells producing relatively high levels of the reporter protein (see below and Fig. 4). Together, these data show that the vector can spread, infect cells, and induce robust transgene expression in an area of more than 3 mm in diameter. This is consistent with data reported by others for smaller vectors such as those based on adeno-associated virus (AAV)^6^.

**Figure 3:**
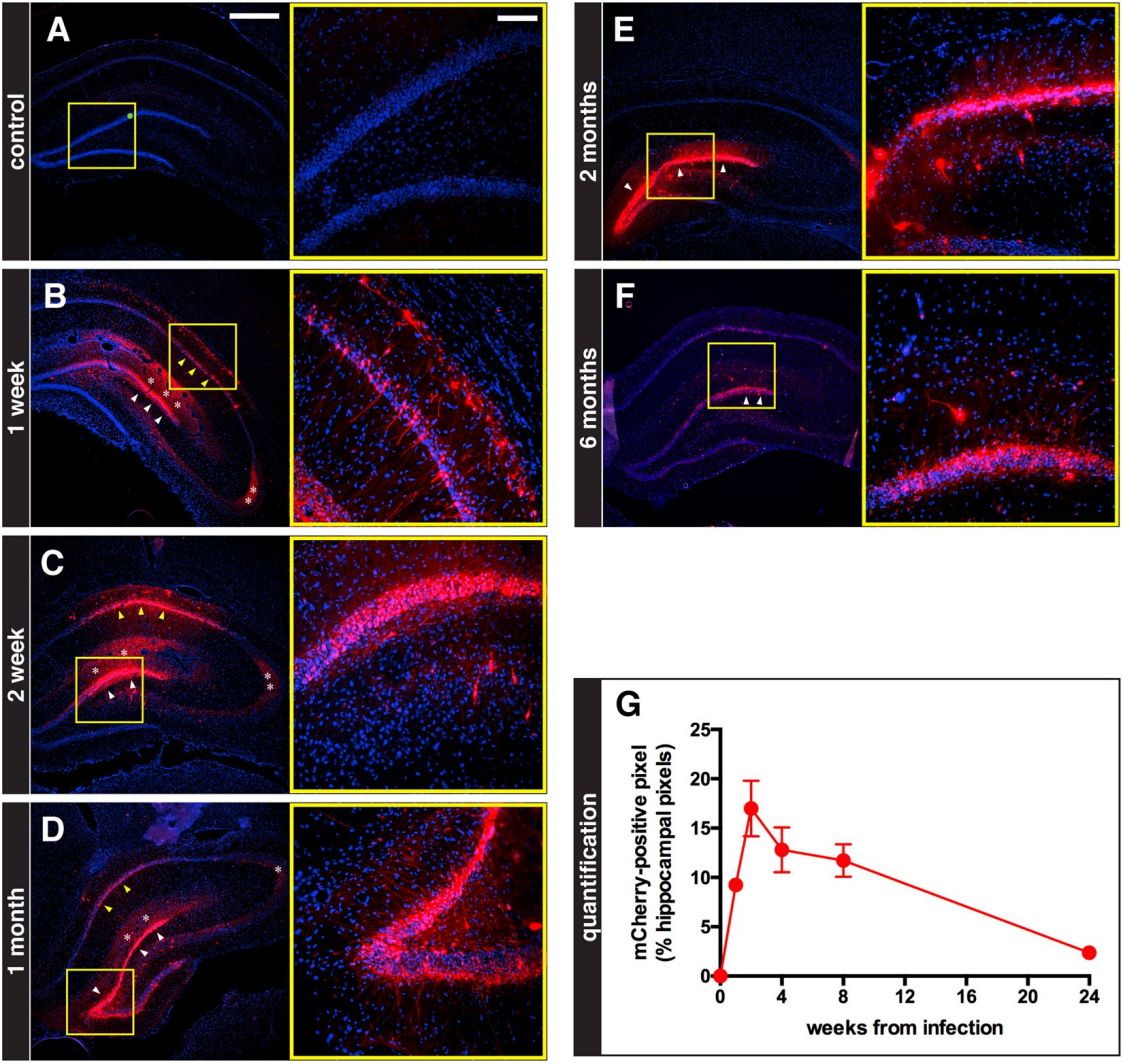
Time course of mCherry expression in the hippocampus. (**A**–**F**) Representative images (5 sections/animal, 4 animals/group) of coronal sections from animals killed at the indicated time-points after JΔNI6 injection into the right hippocampus (see Materials and Methods for details). Left panels show low magnification images, right panels higher magnifications of the areas framed in yellow in the left panels. These sections were at an antero-posterior level immediately adjacent to the site of injection near the upper blade of the dentate gyrus granular layer (needle tip always located at the green spot shown in the left panel of (A). Native mCherry fluorescence in red; nuclei stained in blue with DAPI. Robust mCherry fluorescence is seen in dentate gyrus cells (white arrowheads) and in CA1 neurons (yellow arrowheads); a distinct mCherry signal can be observed in dendrites and axon terminals (white asterisks indicate dentate gyrus granule cell dendrites and terminals). Horizontal bar in (**A**) left panel (for all left panels) = 500 μm; horizontal bar in (**A**) right panel (for all right panels) = 100 μm. (**G**) Quantification of the time-course of native mCherry signal in the hippocampus. Data are the means ± s.e.m. of 4 animals per group.

To determine the time-course of transgene expression, JΔNI6 was injected into the hippocampus as above and animals were killed at different time points to examine eGFP and mCherry fluorescence (Fig. 3). We observed essentially no eGFP fluorescence. This was not due to loss of signal caused by the fixation procedure, because eGFP was easily detected after injection of other backbones and identical fixing procedures. In contrast, mCherry fluorescence was robust, peaking at 2 weeks post injection and remaining high for at least 6 months, the final time-point of the analysis (Fig. 3). A distinct mCherry signal was observed not only in cell bodies, but also in dendrites and axon terminals (Fig. 3C–E). This pattern of expression gradually subsided after 2 weeks, becoming apparently restricted to dentate gyrus cell bodies and proximal dendrites at 6 months.

**Figure 4:**
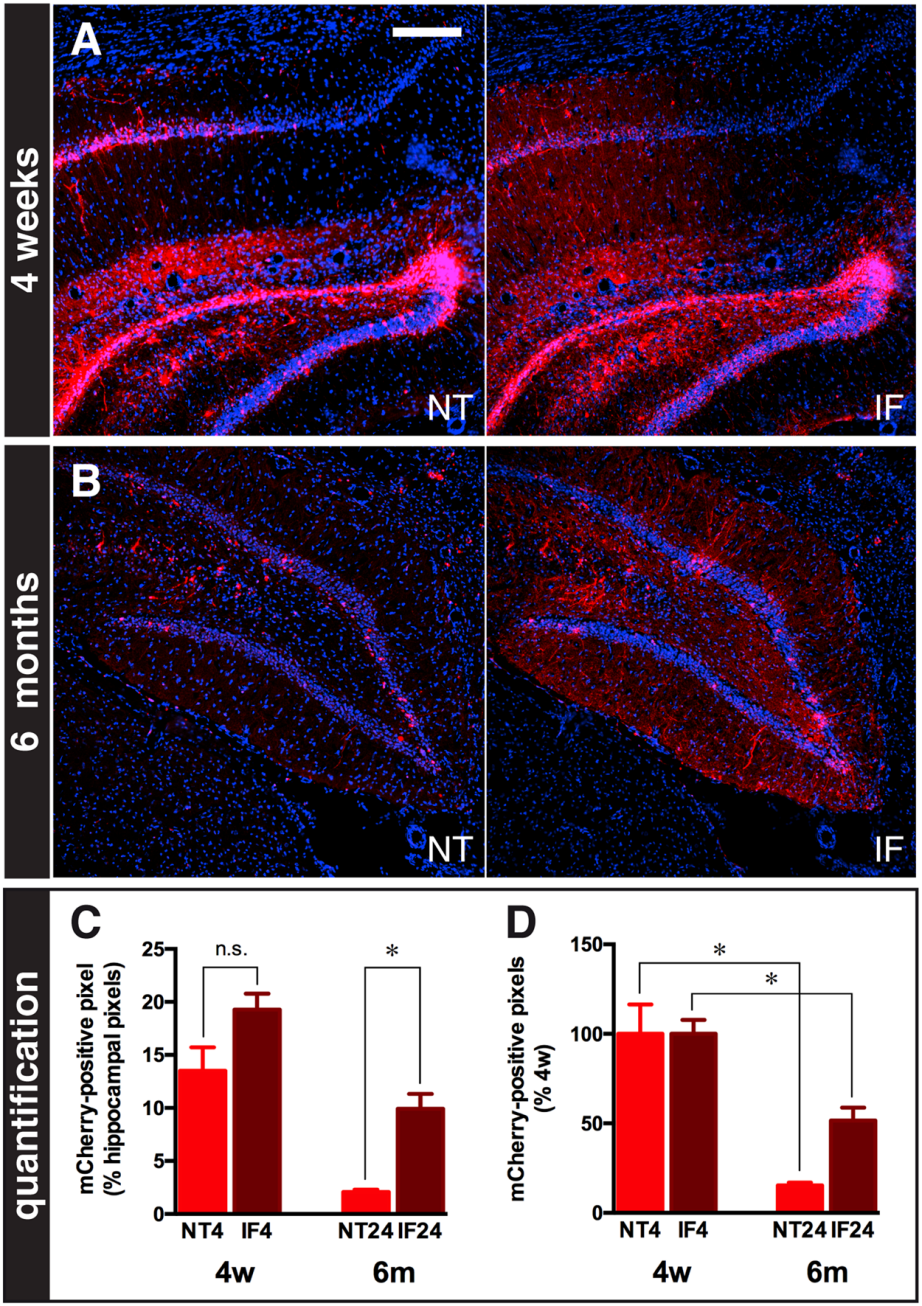
Comparison of native (NT) and immune-fluorescence (IF) mCherry signals. (**A**,**B**) Representative images (5 sections/animal, 4 animals/group) of coronal sections from animals killed 4 weeks (**A**) or 6 months (**B**) after JΔNI6 injection. NT and IF signals, both in red, are shown for the same fields. Nuclei are stained in blue with DAPI. Size bar (all panels) = 100 μm. (**C**) Quantification of NT (bright red) and IF (dark red) mCherry signals at 4 weeks (4 w) and 6 months (6 m). **(D)** Relative decay of NT (bright red) and IF (dark red) mCherry signals between 4 weeks (4 w) and 6 months (6 m). Data are the means ± s.e.m. of 4 animals per group. n.s., not significant; *P < 0.05, Mann-Whitney U test.

A decline in expression levels may be due to vector toxicity (causing death of infected cells) or to reduced levels of transgene expression. In order to address this question and maximize the sensitivity of mCherry detection, we used brain sections from animals sacrificed 4 weeks or 6 months after JΔNI6 injection to compare the pattern of intrinsic mCherry fluorescence with that of mCherry immuno-fluorescence (IF). As expected, the IF signal was greater than the intrinsic signal. However, whereas only a slightly greater signal was observed at 4 weeks, a substantially greater signal was observed at 6 months (Fig. 4A,B). Quantification revealed that the IF signal was approximately 60% greater than the intrinsic signal at 4 weeks, but some 5 times (500%) greater at 6 months (Fig. 4C). Whereas the decay of the intrinsic signal between 4 weeks and 6 months was approximately 85%, the IF signal declined by less than 50% in this interval (Fig. 4D). These data suggest that the reduction in native mCherry fluorescence over time observed earlier was due to reduced levels of transgene expression rather than cell death.

**Figure 5:**
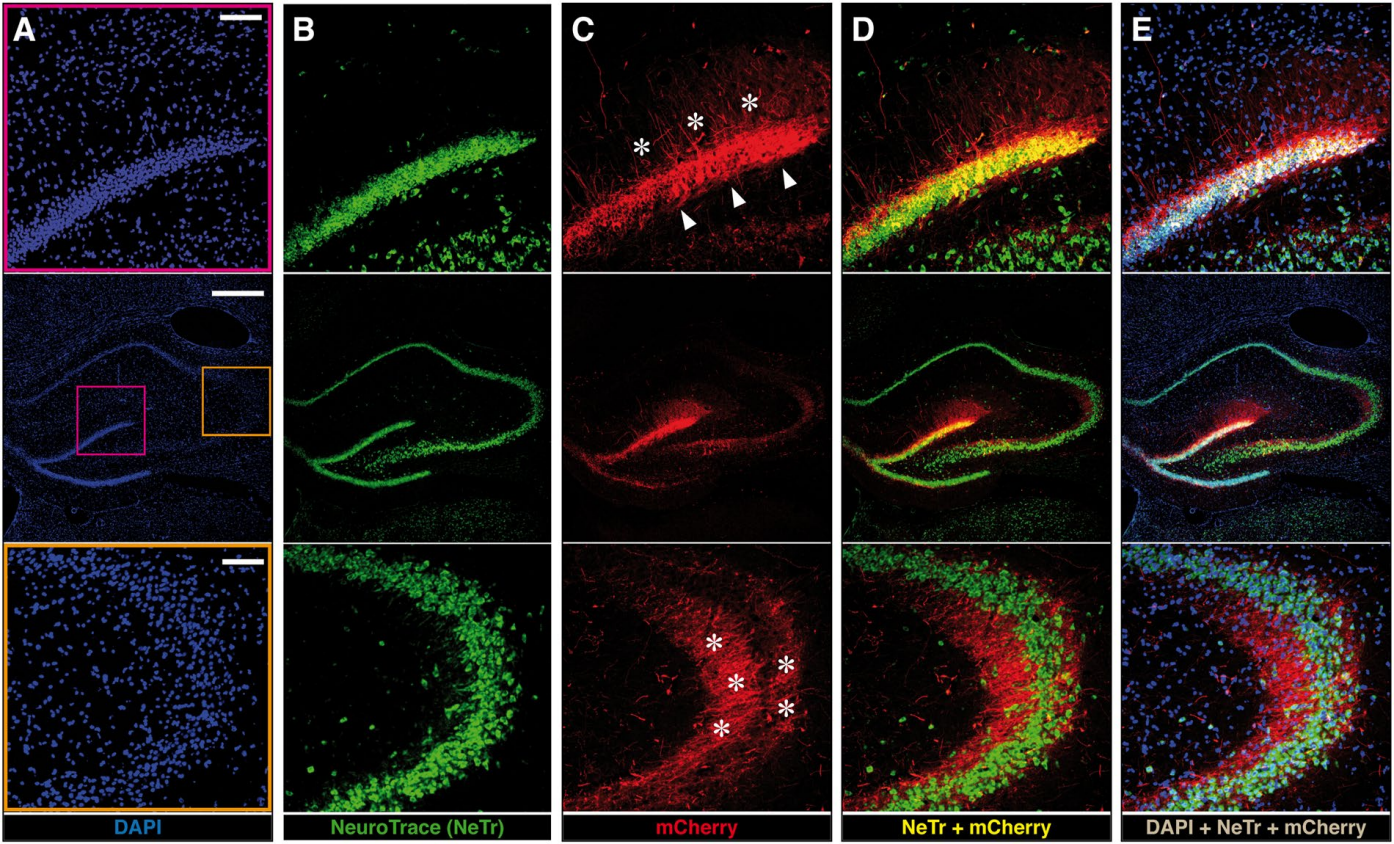
Localization of mCherry expression in the hippocampus. Representative images (5 sections/animal, 4 animals/group) taken from coronal sections prepared from animals killed 2 months after JΔNI6 injection into the right hippocampus. Middle panels show low magnification images of the whole hippocampus; upper panels show higher magnifications of the dentate gyrus including the granular layer (site of granule cell somas), framed in pink in the middle panel A; lower panels show higher magnifications of the CA3 areas (site of the axon terminals originating from the granule cells), framed in orange in the middle panel A. (**A**) Nuclei, stained in blue with DAPI. (**B**) Nuclei of neurons, stained in green with neurotrace. (**C**) Native mCherry florescence (red). Arrowheads, mCherry signals in granule cell bodies; asterisks, mCherry signals in dendrites (upper panel) and axon terminals (lower panel). (**D**) Neurotrace and mCherry double-fluorescence. Note overlapping signal (yellow) in granule cells (upper panel) but not in CA3 pyramidal cells (lower panel). (**E**) DAPI, neurotrace and mCherry triple-fluorescence. Note overlapping signal (white) in granule cells (upper panel) but not in CA3 pyramidal cells (lower panel). Also note that non-neuronal cells (blue nuclei) do not express mCherry. Horizontal bar in (**A**) (middle panel), 500 μm; horizontal bar in (**A**) (upper and lower panels), 100 μm.

To identify the cell types in which the vector could induce transgene expression, we performed double staining with NeuroTrace, a method for visualizing nuclei and soma of neurons based on a fluorescent dye selective for the Nissl substance of neurons. In sections prepared from animals killed 2 months after JΔNI6 injection into the dorsal hippocampus, we observed overlapping mCherry and NeuroTrace signal in granule cells but not in CA3, where mCherry was in granule cell terminal axons (the mossy fibers) but not in pyramidal cell somas (Fig. 5). Essentially all mCherry-positive cells were NeuroTrace positive, indicating that neurons were the prevalent cell type expressing the transgene (Supplementary Fig. S1).

**Figure 6:**
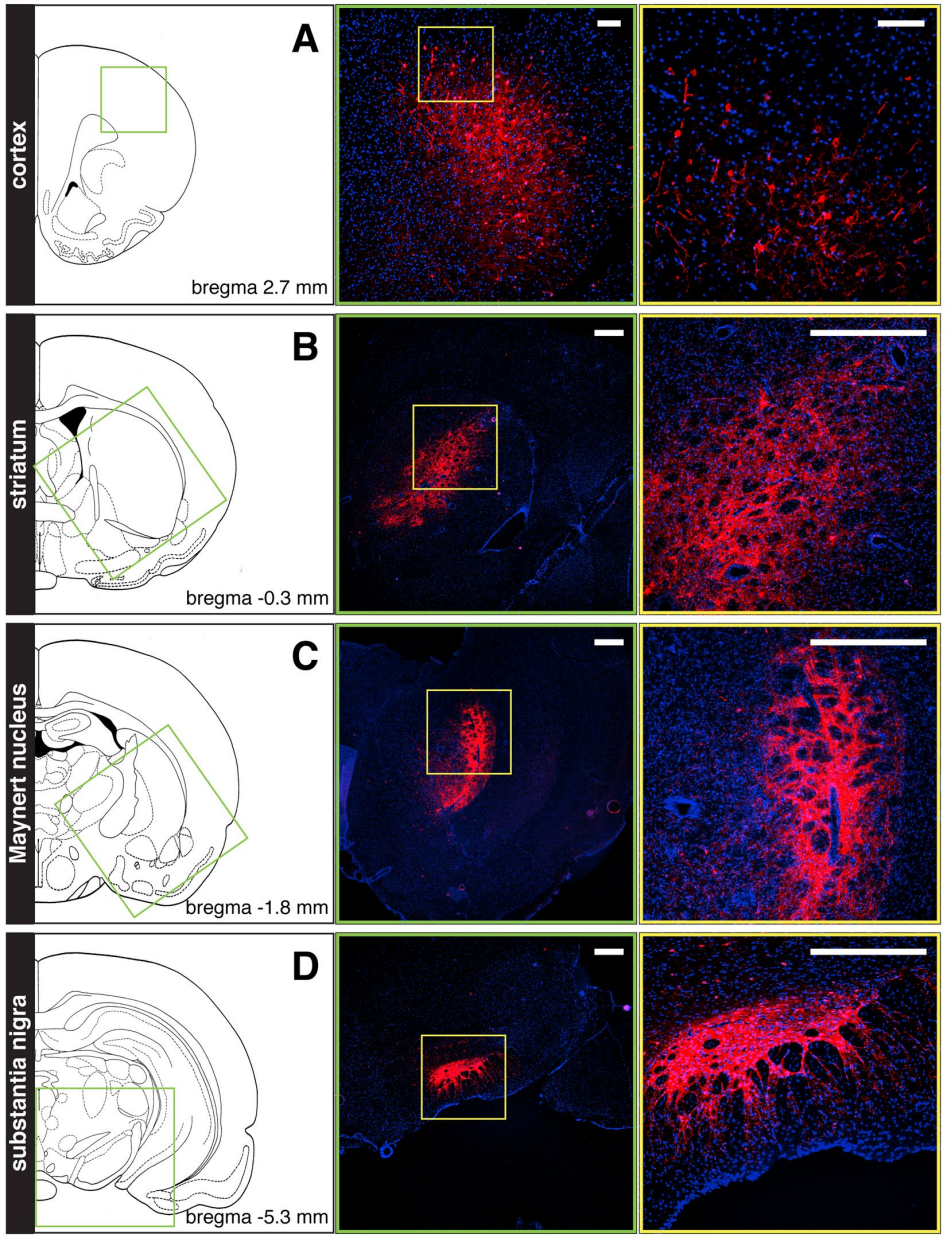
Transgene expression in different brain areas. Representative images (5 sections/animal, 4 animals/group) taken from coronal sections prepared from animals killed 2 weeks after JΔNI6 injection into the prefrontal cortex (**A**), striatum (**B**), Maynert nucleus (**C**) or substantia nigra (**D**). Native mCherry fluorescence in red; DAPI-stained nuclei in blue. Left panels are schematic drawings (adapted from Paxinos and Watson^22^ with permission from Elsevier) of the brain structures at the level of vector injection; anteroposterior coordinates relative to bregma are indicated. Central panels are images of sections corresponding to the area framed in green in the corresponding left panel. Right panels are higher magnifications of the areas framed in yellow in the corresponding central panel. Horizontal bars in **A** = 100 μm; horizontal bars in **B**–**D** = 500 μm.

Finally, we analyzed mCherry expression following injection of JΔNI6 in other brain areas. We chose areas that are thought to play key roles in neurological and psychiatric disorders: the prefrontal cortex (schizophrenia, Alzheimer’s disease), striatum (Huntington’s and Parkinson’s disease), Maynert nucleus (Alzheimer’s disease) and substantia nigra (Parkinson’s disease). Robust transgene expression was observed in each of these areas (Fig. 6), indicating that the JΔNI6 backbone may have utility for multiple disease conditions.

### Safety

**Figure 7:**
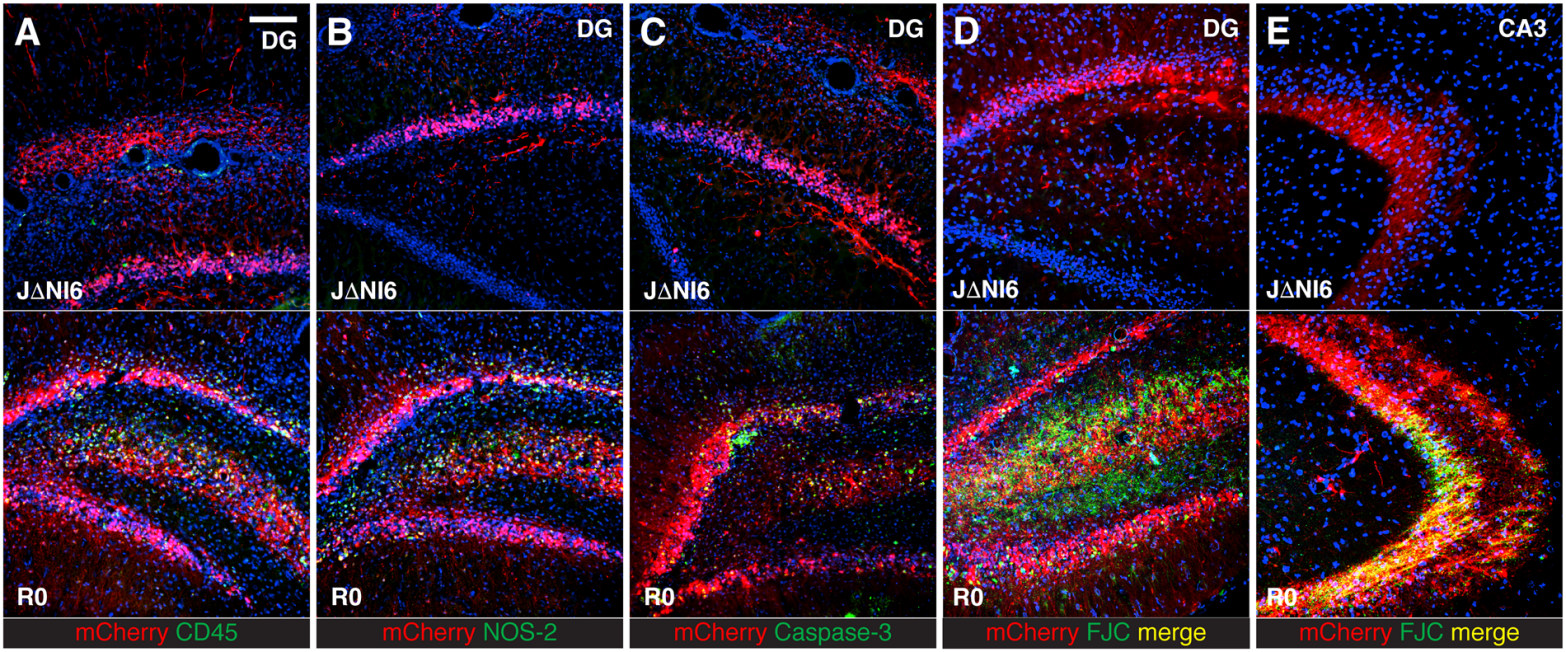
Safety of the JΔNI6 vector. Comparative effect of the JΔNI6 vector (upper panels) and the R0 vector (lower panels) on lymphocyte infiltration detected with anti-CD45 antibody (**A**), oxidative stress detected with anti-NOS-2 (**B**), apoptosis detected with anti-activated caspase 3 (**C**), and neuronal damage detected using fluoro-jade C (FJC, **D**–**E**). Animals (4 per group) were killed 1 week after vector injection into the hippocampus, and images were taken at the level of the dentate gyrus (DG), near the site of vector injection (**A**–**D**) and in the CA3 area, site of axon terminals from dentate gyrus granule cells (**E**). Native mCherry fluorescence in red; nuclei stained in blue with DAPI; CD45, NOS-2, caspase-3 and FJC staining in green in panels (A,B,C, and D, E), respectively. Horizontal bar in (**A**) (for all panels) = 100 μm.

Since previous generations of HSV vectors were hampered by immunogenicity and toxicity to infected cells, we evaluated these responses to JΔNI6 inoculation. It is well documented that ICP0 is a leading cause of replication-defective HSV-1 cytotoxicity^7, 8^, and we therefore rescued ICP0 in a closely related vector without eGFP gene (JΔNI5)^2^ to serve as a positive experimental control for the detection of vector toxicity (JΔNI5R0 vector). We tested lymphocyte infiltration, oxidative stress and apoptosis in animals killed one week after vector injection by staining for appropriate markers, respectively CD45, inducible nitric oxide synthase (iNOS or NOS-2) and activated caspase 3. This early time-point was chosen because these different adverse events tend to become less pronounced during the second week after injection of previous-generation vectors^9^, observations that we confirmed in preliminary experiments with the JΔNI5R0 vector. Our results at 1 week post vector injection showed many marker-positive cells in brains injected with JΔNI5R0, whereas brains injected with JΔNI6 were almost completely negative (Fig. 7A–C). In addition, we examined neuroinflammatory effects by analyzing microglia activation using Iba1 immunostaining. Microglia activation was observed 1 week post JΔNI5R0 injection, but not after injection of JΔNI6 (Supplementary Fig. S2). Activation was no longer observed at 1 month.

Finally, we explored neuronal damage using fluoro-jade C (FJC), a fluorescent dye that labels degenerating neurons. Again, these experiments were performed in animals killed one week after vector injection into the hippocampus and in comparison with JΔNI5R0. Of note, the JΔNI5R0 virus in this and the previous experiment was deleted for the BAC region while JΔNI6 was not; we previously reported that removal of the BAC region eliminated residual toxicity of our IE gene-depleted vectors for human fibroblasts in culture^2^. Whereas many FJC-positive cells were observed in brains injected with JΔNI5R0, virtually none were detected in JΔNI6-injected brains (Fig. 7D,E). We cannot fully explain the observation of FJC-positive cells and elongations that were not mCherry positive, but these may reflect indirect damage by JΔNI5R0 caused by lymphocyte infiltration or oxidative stress. In time, neuronal damage induced by JΔNI5R0 resulted in a dramatic cell loss with obvious hippocampal degeneration at 1 month, whereas no hippocampal shrinkage was observed with JΔNI6 (Supplementary Fig. S3). Together, these data support the notion that JΔNI6, even with an intact BAC region, is devoid of detectable immunogenicity and toxicity to infected cells.

## Discussion

Several hurdles must be overcome before gene therapy becomes a standard procedure for the treatment of neurological diseases. Indeed, while therapeutic gene transfer with AAV and lentivirus (LV) vectors has recently enjoyed considerable success for the treatment of peripheral diseases, to the point that some of these have gained approval for clinical use in Europe (Glybera), gene therapies for the central nervous system (CNS) have proved more difficult. First, they often require the delivery of large genes or complex multigene cassettes that greatly exceed the capacity of LV and AAV vectors^1^. To accommodate larger payloads, larger viruses such as adenovirus (AdV) and HSV would be required. Although AdV vectors have limited capacity, “gutted” derivatives can incorporate up to 30 kb of foreign sequences^10^. However, gutted AdV vectors are typically contaminated with small percentages (but large particle numbers) of helper virus, and these contaminants engender antiviral responses that limit transgene expression, cause local inflammation and induce antiviral immunity impeding vector re-administration. Second, the ideal CNS vector should deliver to specific cell types after being injected into a highly heterogeneous tissue, since failure to do so can lead to adverse or paradoxical effects^11^. Third, vectors whose genomes integrate into the host cell DNA can lead to transformation events that may result in harm to the patient.

Vectors derived from AAV, LV or AdV do not combine all features necessary for CNS applications. In principle, HSV is an attractive alternative, having a genome of 152 kb, ~25 kb of which can be deleted to accommodate up to 30 kb of foreign sequences. Moreover, the virus genes are expressed in a cascade fashion, such that deletion of the first expressed IE genes completely shuts down viral gene expression and replication. Finally, HSV does not integrate its DNA into the host cell genome like LV vectors, eliminating the risk of insertional mutagenesis. In spite of these advantages, HSV has thus far been relatively overlooked because of concerns over cytotoxicity, immunogenicity, and difficulty in achieving persistent expression in the CNS. The new generation of HSV vector designs described here and elsewhere^2^ overcomes these problems while maintaining the ability to host large payloads of non-viral genes.

It has previously been shown that inactivation of all IE genes eliminates HSV cytotoxicity^12–14^. The present study expands these observations to the brain, documenting the absence of neurotoxicity and lymphocyte infiltration while confirming that ICP0 is a key inducer of both. Although amplicon vectors do not express any HSV gene, they are still toxic and pro-inflammatory, likely due to contamination with a low percentage but large number of helper viruses expressing ICP0 and other toxic IE proteins^15^. Elimination of ICP0 has proven problematic, however, since ICP0 counter-acts heterochromatinization of the viral genome^4^ and is thereby essential for the maintenance of transgene expression in most cell types. We recently reported that genetic elements associated with the LAT gene can compensate to a degree for the absence of ICP0 in non-toxic vectors by protecting an embedded reporter gene from rapid silencing in non-neural cells^2^, potentially by blocking heterochromatin encroachment. In the same study, we noticed that transgenes outside this protective environment, while silent in most cells, were active in sensory neurons for several days. The essential observation in the present study is that a reporter expression cassette in the ICP4 locus escaped global silencing of an IE gene-depleted HSV genome in CNS neurons for at least 6 months. A second reporter gene was silent throughout and we observed no overt signs of toxicity or an immune response in vector-injected animals, arguing against genome-wide transcriptional derepression. These results raise the expectation that a safe and effective vector system for the delivery of large or multiple therapeutic transgenes for CNS disorders is within reach.

At this time we do not have a mechanistic explanation for the observation of sustained expression of mCherry, but not eGFP, in JΔNI6-infected CNS neurons. While potential differences in the stabilities of mCherry and eGFP mRNA and protein in these cells could be contributing factors, the more plausible hypothesis may be that the choice of transgene promoter and/or insertion site are critical for sustained expression. Further studies are ongoing to distinguish between these alternative possibilities.

Our data indicate that the mCherry gene was expressed in neurons, but not in astrocytes. Co-visualization of neurons (using NeuroTrace) and mCherry fluorescence showed hundreds of double-positive cells per field, but no mCherry-positive, NeuroTrace-negative cells. This result suggests that mCherry expression in astrocytes was at best orders of magnitude lower than in neurons. It will be of interest to determine whether this is a function of the transgene promoter (see below).

We found that JΔNI6 can induce transgene expression in multiple brain areas that are relevant to neurological diseases, including Parkinson’s, Alzheimer’s and several forms of epilepsy. The dramatic difference in both the level and duration of neuronal expression of the two transgenes of JΔNI6 raises several questions: (1) What is responsible for these differences, the promoter, the location of the expression cassette in the viral genome, or both? (2) If, as we suspect, location of the expression cassette is important, are there neuron-specific anti-silencing elements in the vicinity of the ICP4 locus? These may include regions enriched in CTCF-binding motifs flanking the ICP4 deletion in JΔNI6 on both sides^2, 16^ or yet unknown sequences. Of interest, it has been reported that transcriptional activity from an IE gene-depleted vector tends to be higher in neuronal cells in the repeat segments of the HSV genome than in the unique regions^17^, but the mechanism is unknown. Examination of transgene expression from other loci in the viral repeats may reveal whether the sustained mCherry expression we see is a unique property of the ICP4 locus or more generally of locations with the repeats. (3) Can transgene expression from the ICP4 locus be switched to a different cell type using a cell-type-specific promoter or will this require additional manipulations to protect the site from heterochromatinization? Answers to these questions may provide the framework to expand the utility of our vectors to other cells of the CNS.

Our vector injection technique accomplished relatively broad transgene expression, spanning a distance of more than 3 mm across the injection point. It is likely that the vector spreads even more broadly because our distance estimate was based on detection of intrinsic mCherry fluorescence, i.e., of cells expressing the transgene at high levels; we have shown that additional infected cells can be detected by immunohistochemistry. While vector spread in the range we observed is perfectly suitable for studies in small rodents, translation to human diseases will require scale-up modeling in larger animals, such as mini-pigs or primates. Options are to design needles with multiple holes, to increase volumes and titers of vector injected, and/or to use convection-enhanced delivery systems for infusion^18, 19^. Moreover, it may be possible to increase the efficiency of vector delivery to neurons by viral glycoprotein engineering to avoid virion attachment to other cells^20^.

In conclusion, we report here a new generation of vectors that overcome the major limitations of HSV as a gene delivery vehicle for CNS disorders, namely short-term transgene expression and toxicity, while maintaining its advantages, in particular the ability to host large and/or multiple transgenes. These features represent a pivotal step forward in the development of vectors capable of selective expression of a single large therapeutic gene or entire regulatory networks in CNS neurons. Before first-in-man clinical experiments, however, remaining hurdles must be overcome. Aside from the need to increase virus spread in the injected brain, these include potential responses to re-dosing and inoculation of immunized individuals. Assuming these hurdles can be overcome in the future, our vectors may prove valuable for therapeutic intervention in the variety of diseases of the CNS that require the expression of large and/or multiple therapeutic transgenes. In parallel, a better understanding of the mechanism(s) that allow persistent JΔNI6 reporter gene expression in neurons may lead to further improvements in vector design to benefit the treatment of CNS pathology.

## Methods

### Vector engineering

JΔNI6, previously referred to as JΔNI6GFP, was as described^2^. JΔNI5R0 was derived from JΔNI5, which is JΔNI6 without the eGFP expression cassette. A 7.5-kb *Dra*I-*Psi*I fragment spanning the ICP0 locus deletion in JΔNI5 was isolated from purified KOS-37 BAC DNA and cloned into pCRBlunt to generate plasmid pCRBluntICP0. The kanamycin-resistance gene flanked by an I-*Sce*I restriction site [I-*Sce*I-*aphAI* fragment^2^] was amplified from pEPkan-S2^21^ [kindly provided by N. Osterrieder, Free University of Berlin, Berlin, Germany] by PCR with primers R0f and R0r specified below, providing terminal *Mfe*I restriction sites, and the *Mfe*I-digested product was cloned into the unique *Mfe*I site of pCRBluntICP0. The complete pCRBlunt insert was isolated and recombined with JΔNI5 DNA by Red-mediated recombination in *Escherichia coli*, essentially as described^2, 21^, and the *aphAI* gene was removed by I-*Sce*I induction, also as described^2, 21^. Accurate repair of the ICP0 locus was confirmed by field inversion gel electrophoresis of restriction enzyme-digested BAC DNA, diagnostic PCR, and targeted DNA sequencing. Recombinant BAC DNA was converted to infectious virus by transfection of U2OS-ICP4/27 cells^2^. ICP0 expression was confirmed by Western blot analysis of infected U2OS cells.

*Primer R0f*:

5′-TATCAATTGCGCAACACCTGCCCGCTGTGCA ACGCCAAGCTGGTGTACCTGATAGTGGGAGGATGACGACGATAAGTAGGGATA-3′

*Primer R0r*:

5′-AATCTGCAGCAATTGCTACAACCAATTAACCAATTCTGATTAG-3′

### Virus production

HSV vectors were propagated on U2OS-ICP4/27 cells in T150 tissue culture flasks. Twenty-four h before infection, cells were seeded as a 50% confluent monolayer to reach a confluence of 90–100% the following day. Cells were infected in serum free medium at a multiplicity of 0.0001 plaque-forming units (pfu) per cell and incubated at 33 °C in 5% CO_2_. After 5–7 days, the supernatant was collected and sequentially filtered through a 0.8 and a 0.45 μm nitrate cellulose membrane, and the virus was concentrated by high-speed centrifugation (45 min at 19,000 rpm in a JA-20 rotor). The viral pellet was re-suspended in phosphate-buffered saline (PBS) and stored at −80 °C in small aliquots. The JΔNI5R0 virus used in this study was deleted for the BAC region by passage through U2OS-ICP4/27-Cre cells, as described^2^.

### Virus titration by qPCR

Viral DNA was extracted with the DNeasy Blood and Tissue Kit (Qiagen, Hilden, Germany) according to the manufacturer’s instructions. Quantitative (q) PCR was performed using the BioRad (Hercules, California, USA) CFX PCR System with customized primers and probe (BioRad) specific for the HSV glycoprotein D (gD) gene^2^. Viral genome copies were quantified by interpolation of a standard curve generated by tenfold serial dilution of a gD gene-containing plasmid of known concentration.

### *In vivo* HSV injection

Under ketamine (90 mg/kg i.p.) and xylazine (13 mg/kg i.p.) anesthesia, a total of ~2.5 × 10^8^ viral genome copies were inoculated into the selected brain regions of Sprague Dawley male rats (300 g; Harlan, San Pietro al Natisone, Italy). The stereotactic coordinates for inoculation, based on the Paxinos atlas^22^, were as follows (0 bregma): dorsal hippocampus AP −3.5, ML +2.1, DV −3.5; prefrontal cortex AP +3, ML +2.5, DV −3; striatum AP +1.5, ML −2.7, DV −5.5; nucleus basalis of Maynert AP −1.3, ML +2.4, −7; substantia nigra AP −4.8, ML −2.4, DV −8.2. The viral vectors were injected by stereotactic implantation of a borosilicate glass needle, custom-made with a laser microdissector (CTR6000; Leica Microsystems, Wetzlar, Germany). The needle tips were chamfered through laser cutting (angle: 45 degrees; inner diameter at the tip: 60 μm; outer diameter at the tip: 80 μm) and an additional hole (diameter 10 μm) was opened 100 μm above the tip, to favor the spread of the injected solution. The needle was bottom-filled with the viral prepatration and linked to a microperfusion pump, in order to inject a total of 3 μl solution at a flow rate of 0.2 μl/min. These experiments were performed at the University of Ferrara in compliance with the guidelines for the ethical treatment of experimental animals (authorization from the Italian Ministry for Health D.M. 246/2012-B).

### Tissue preparation

At the selected time points following vector injection, animals were deeply anesthetized with pentobarbital and perfused with 4% paraformaldehyde in 0.1 M PBS. Brains were isolated and postfixed in 4% paraformaldehyde for 1 h, then cryoprotected in 30% sucrose at 4 °C until tissue sank, and finally flash-frozen in isopentane chilled to −80 °C.

### Native mCherry imaging and quantification

Frozen brains were cut into 20 μm thick coronal sections in a cryostat (Leica Microsystems). For direct imaging of native mCherry fluorescence, slices were immediately counterstained for nuclei with Pro-Long Gold antifade reagent with 4′,6-diamidino-2-phenylindole (DAPI; Thermo Fisher, Waltham, MA, USA) and mounted on microscope glass slides for observation. Image analyses were conducted using a DMRA2 microscope (Leica Microsystems), equipped with the Metamorph Image Analysis software (Universal Imaging Inc., Downingtown, PA, USA).

### Immunofluorescence

Twenty μm coronal sections were cut at −20 °C in a cryostat, rinsed in 0.1 M PBS and blocked in 0.1 M PBS with 10% normal goat serum and 0.3% Triton X-100. Sections were then incubated overnight at 4 °C with primary antibodies dissolved in blocking solution: rabbit anti-mCherry (Thermo Fisher, 1:100); rabbit anti-cleaved caspase-3 (Cell Signaling, Danvers, MA, USA, 1:100); rabbit anti-nitric oxide synthase-2 (Santa Cruz, Dallas, TX, USA, 1:100); mouse anti-CD45 (Santa Cruz, 1:100); rabbit anti-Iba1 (Synaptic Systems, Gottingen, Germany, 1:200). The next day, sections were incubated with secondary antibodies for 2 h at room temperature, as follows: Alexa Fluor 488 goat anti-rabbit (Molecular Probes, Eugene, OR, USA), 1:1,000; Alexa Fluor 488 goat anti-mouse (Molecular Probes), 1:1,000. In addition, 10 μM DAPI (Molecular Probes) was added to the secondary antibody solution in order to label cell nuclei.

### NeuroTrace staining

After rehydration in 0.1 M PBS, sections were permeabilized with 0.1% Triton X-100 in PBS for 10 min, washed twice for 5 min in PBS and stained with NeuroTrace 640/660 Deep Red Fluorescent Nissl stain (ThermoFisher; dilution 1:200), for 20 min at room temperature. Sections were then washed in PBS and 0.1% Triton X-100, twice with PBS, and finally left for 2 h at room temperature in PBS before imaging.

### FluoroJade-C staining

Cryostat brain sections were mounted on frosted microscope slides and pretreated for 5 min by immersion in 80% EtOH and 1% NaOH, followed by 2 min in EtOH 70% and 2 min washing in distilled water. These sections were then incubated for 10 min in 0.06% KMnO_4_ and rinsed in distilled water for 3 min, before incubation for 10 min in a solution of 0.0001% FluoroJade-C (Immunological Sciences, Rome, Italy) and 0.1% acetic acid. After three washes in distilled water for 1 min, slides were dried, dehydrated with xylene, and coverslipped with DPX mountant for microscopy (Sigma, Saint Louis, MO, USA).

### Quantification and statistical analysis

Images of the hippocampus were captured using a Hamamatsu C11440 camera (Hamamatsu, Japan) mounted on a Leica DMRA2 microscope, in 2^16^ gray levels. Using the Metamorph software, the area of the hippocampus was selected as the region of interest (ROI) and the minimum and average gray levels within the ROI were calculated. mCherry-positive pixels were identified by thresholding at the gray level corresponding to the mean plus the difference between average and minimum. Using this approach, only those pixels that were significantly above background (i.e., mCherry-positive) were selected. Data were then expressed as percent of mCherry-positive pixels over total hippocampal pixels. Five sections per animal were analyzed, one in every five 20 μm sections cut across the injection site, i.e., one every 100 μm with the 3^rd^ at the site of injection. Numbers from these 5 sections were used as quintuplicates, i.e., the average was used for statistical analysis. Confocal images were captured using a HAL 100 camera (Zeiss, Oberkochen, Germany) mounted on a Zeiss LSM510 confocal microscope.

Statistical analysis of the comparison between native and immuno-fluorescence mCherry signal was performed using a non-parametric test (Mann-Whitney U test for unpaired data).

## Electronic supplementary material

Supplementary Figures S1-S3

## References

[1] Simonato M (2013). Progress in gene therapy for neurological disorders. Nat Rev Neurol.

[2] Miyagawa Y (2015). Herpes simplex viral-vector design for efficient transduction of nonneuronal cells without cytotoxicity. Proc Natl Acad Sci USA.

[3] Ferenczy MW, DeLuca NA (2011). Reversal of heterochromatic silencing of quiescent herpes simplex virus type 1 by ICP0. J Virol.

[4] Boutell C, Everett RD (2013). Regulation of alphaherpesvirus infections by the ICP0 family of proteins. J Gen Virol.

[5] Uchida H (2010). A double mutation in glycoprotein gB compensates for ineffective gD-dependent initiation of herpes simplex virus type 1 infection. J Virol.

[6] Woldbye DP (2010). Adeno-associated viral vector-induced overexpression of neuropeptide Y Y2 receptors in the hippocampus suppresses seizures. Brain.

[7] Samaniego LA, Wu N, DeLuca NA (1997). The herpes simplex virus immediate-early protein ICP0 affects transcription from the viral genome and infected-cell survival in the absence of ICP4 and ICP27. J Virol.

[8] Krisky DM (1998). Deletion of multiple immediate-early genes from herpes simplex virus reduces cytotoxicity and permits long-term gene expression in neurons. Gene Ther.

[9] Paradiso B (2009). Localized delivery of fibroblast growth factor-2 and brain-derived neurotrophic factor reduces spontaneous seizures in an epilepsy model. Proc Natl Acad Sci USA.

[10] Altaras NE (2005). Production and formulation of adenovirus vectors. Adv Biochem Eng Biotechnol.

[11] Haberman R (2002). Therapeutic liabilities of *in vivo* viral vector tropism: adeno-associated virus vectors, NMDAR1 antisense, and focal seizure sensitivity. Mol Ther.

[12] Jackson SA, DeLuca NA (2003). Relationship of herpes simplex virus genome configuration to productive and persistent infections. Proc Natl Acad Sci USA.

[13] Samaniego LA, Neiderhiser L, DeLuca NA (1998). Persistence and expression of the herpes simplex virus genome in the absence of immediate-early proteins. J Virol.

[14] Terry-Allison T, Smith CA, DeLuca NA (2007). Relaxed repression of herpes simplex virus type 1 genomes in Murine trigeminal neurons. J Virol.

[15] Suzuki M, Chiocca EA, Saeki Y (2008). Stable transgene expression from HSV amplicon vectors in the brain: potential involvement of immunoregulatory signals. Mol Ther.

[16] Amelio AL, McAnany PK, Bloom DC (2006). A chromatin insulator-like element in the herpes simplex virus type 1 latency-associated transcript region binds CCCTC-binding factor and displays enhancer-blocking and silencing activities. J Virol.

[17] Harkness JM, Kader M, DeLuca NA (2014). Transcription of the herpes simplex virus 1 genome during productive and quiescent infection of neuronal and nonneuronal cells. J Virol.

[18] Bankiewicz KS (2000). Convection-enhanced delivery of AAV vector in parkinsonian monkeys; *in vivo* detection of gene expression and restoration of dopaminergic function using pro-drug approach. Exp Neurol.

[19] Kells AP (2009). Efficient gene therapy-based method for the delivery of therapeutics to primate cortex. Proc Natl Acad Sci USA.

[20] Laquerre S (1998). Heparan sulfate proteoglycan binding by herpes simplex virus type 1 glycoproteins B and C, which differ in their contributions to virus attachment, penetration, and cell-to-cell spread. J Virol.

[21] Tischer BK, von Einem J, Kaufer B, Osterrieder N (2006). Two-step red-mediated recombination for versatile high-efficiency markerless DNA manipulation in Escherichia coli. Biotechniques.

[22] Paxinos, G. & Watson, C. *The Rat Brain In Stereotaxic Coordinates* (Academic Press, 1982).

[23] Amaral, D. G. & Witter, M. P. In *The Rat Nervous System* (ed. Paxinos, G.) 443–493 (Academic Press, 1995).

