# Supplementary material for: Engineered HSV vector achieves safe long-term transgene expression in the central nervous system
Source: Sci Rep. 2017 May 4;7:1507. doi: 10.1038/s41598-017-01635-1 (PMC5431452; doi:10.1038/s41598-017-01635-1)
Supplement: Supplementary file 1 — Supplementary Figures S1-S3 [file 41598_2017_1635_MOESM1_ESM.pdf]

Supplementary information

Engineered HSV vector achieves safe long-term transgene expression in the central nervous system

Gianluca Verlengia, Yoshitaka Miyagawa, Selene Ingusci, Justus B. Cohen, Michele Simonato, and Joseph C. Glorioso

Contents

- Supplementary Figures S1-S3

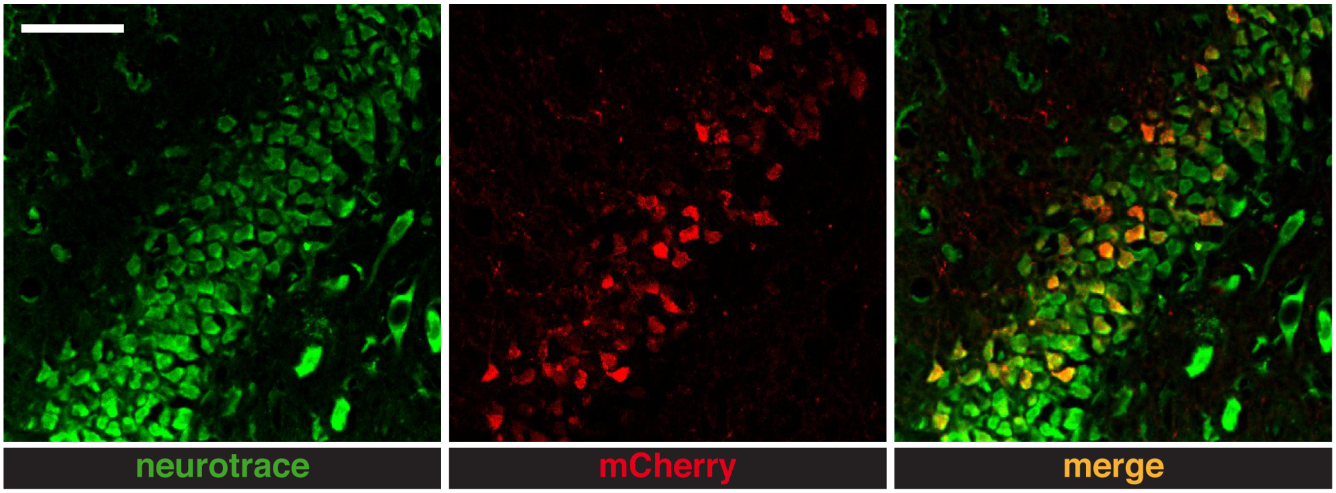

Supplementary Fig. S1. Neurons were the prevalent cell type expressing mCherry. Representative confocal images (4 animals/group) taken at the level of the dentate gyrus upper blade from coronal sections prepared from animals killed 2 months after JΔNI6 injection into the right hippocampus. Note overlapping signal (yellow) in granule cells bodies for all mCherry-expressing cells. Horizontal bar, 50 µm.

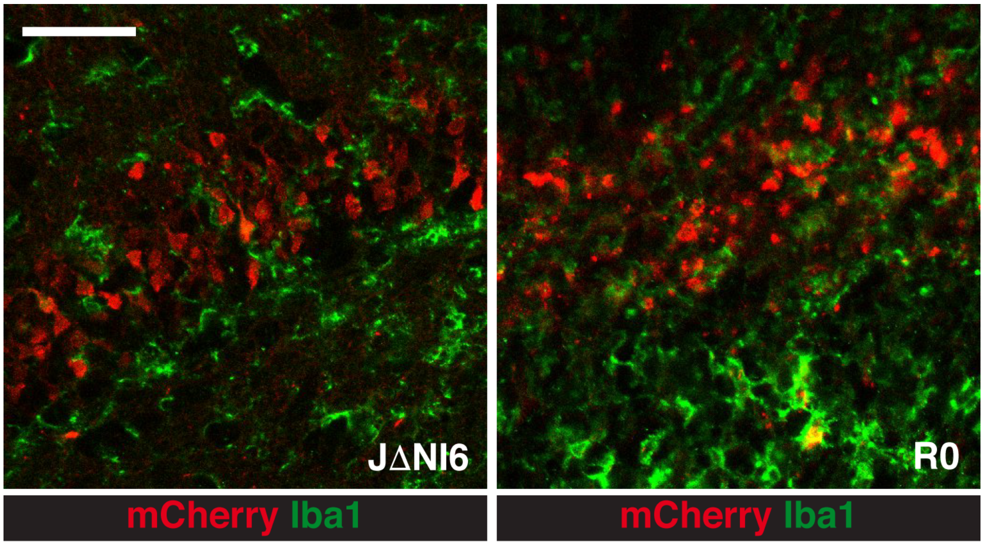

Supplementary Fig. S2. JΔNIR0 causes microglia activation. Representative confocal images (4 animals/group) taken at the level of the dentate gyrus upper blade from coronal sections prepared from animals killed 1 week after JΔNI6 (left) or JΔNI5R0 (right) injection into the right hippocampus. Note resting microglial cells after JΔNI6 injection, and activated microglial cells (i.e., cells with thicker ramifications) after JΔNI5R0 injection. Horizontal bar, 50 µm.

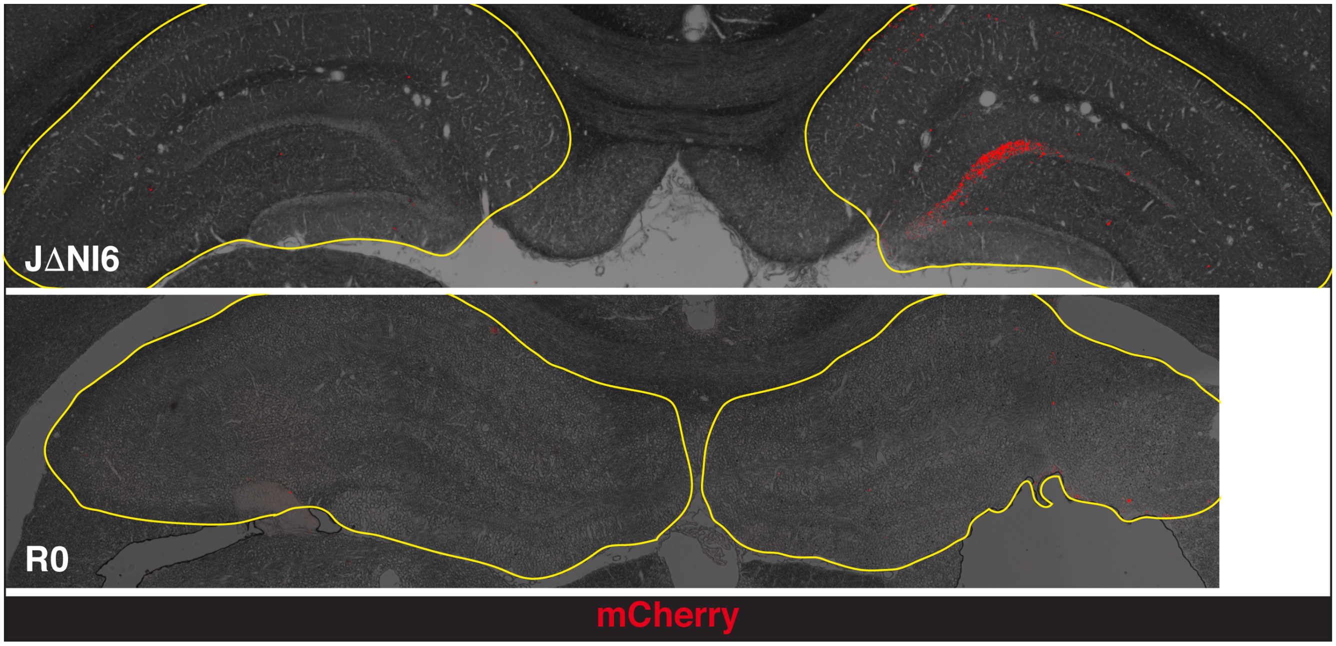

Supplementary Fig. S3. Neuropathological outcome of treatment with vector JΔNI6 (upper panel) or JΔNI5R0 (lower panel) 28 days after injection into the right hippocampus; hippocampi are circled in yellow. JΔNI5R0 causes an obvious reduction in hippocampal size, whereas JΔNI6 does not produce any detectable effect. Note that mCherry expression (red) is robust in the upper panel but is virtually absent in the lower panel, an indication that infected cells died because of vector toxicity. These images are representative of similar findings in 4 other animals per group.
